# Effects of bloom-forming cyanobacterial extracellular polymeric substances on the adsorption of cadmium onto kaolinite: behaviors and possible mechanisms

**DOI:** 10.1186/s40064-016-2191-8

**Published:** 2016-04-27

**Authors:** Xiaolin Kuang, Jihai Shao, Anwei Chen, Si Luo, Liang Peng, Genyi Wu, Ji-Dong Gu

**Affiliations:** College of Resources and Environment, Hunan Agricultural University, Changsha, 410128 People’s Republic of China; Hunan Provincial Key Laboratory of Farmland Pollution Control and Agricultural Resources Use, Hunan Agricultural University, Changsha, 410128 People’s Republic of China; Laboratory of Environmental Microbiology and Toxicology, School of Biological Sciences, The University of Hong Kong, Hong Kong SAR, People’s Republic of China

**Keywords:** *Microcystisaeruginosa*, Extracellular polymeric substances, Cadmium, Kaolinite

## Abstract

Cyanobacterial blooms result in high level of cyanobacterial extracellular polymeric substances (EPS) in water. The effects of bloom-forming cyanobacterial EPS on the distribution of Cd(II) in the interface between sediment and water is unknown. Clay is a main component in sediment. The effects of EPS, originated from a typical bloom-forming cyanobacterium *Microcystisaeruginosa*, on the adsorption and desorption characteristics of Cd(II) by kaolinite were investigated in this study. Results of XRD analysis indicated that cyanobacterial EPS bound on the surface of kaolinite. The composite of kaolinite + EPS showed higher adsorption capacity toward Cd(II) than pure kaolinite, and hydroxyl groups were involved in the adsorption processes. The data for the adsorption of Cd(II) by kaolinite are well fitted by both Langmuir model and Freundlich model, whereas only Freundlich model well describes the adsorption data of Cd(II) by the composite of kaolinite + EPS. The adsorption of Cd(II) onto kaolinite was an exothermic process, but it became an endothermic process after EPS incorporation. Results of desorption showed that EPS incorporation increased the adsorption of kaolinite toward Cd(II) through physical adsorption, ion exchange and complexation.

## Background

Heavy metal ions are toxic, non-biodegradable and can accumulate through food chain. Heavy metal pollution has caused serious ecological problems and posed a great health risk to human. Cadmium is a most hazardous heavy metal due to its high toxicity and carcinogenic effects (Son et al. 2012). In recent decades, industrial effluent and agricultural run-off caused widespread cadmium contamination in aquatic environments (Öztürk et al. 2009; Florian et al. 2011).

Another world wide water problem is the eutrophication and harmful algal blooms. *Microcystis* is a typical bloom-forming cyanobacterium. It frequently dominates in eutrophic fresh waters. Take China as an example, two large lakes, Lake Taihu and Lake Dianchi, were all received severe *Microcystis* based water blooms (Ye et al. 2009; Wu et al. 2014). *Microcystis* could maintain very high cell density in water during bloom formation stage. Ye et al. (2009) reported that the total cyanobacterial density (mainly as *Microcystis*) reached as high as 2.93 × 10^11^ cells/mL in Lake Taihu, China. *Microcystis* can excrete EPS into water. High cell density of *Microcystis* frequently results in high concentration of EPS in water column. Xu et al. (2013) reported that the EPS in cultures of *Microcystisaeruginosa* researched 130 μg per 10^7^ cells. The main component of EPS in *Microcystis* culture is polysaccharides, and then followed by proteins (Xu et al. 2013). EPS enrich hydroxyl groups, carboxylic groups, acetylated amino, and also contain some noncarbohydrate constituents, e.g. phosphate and sulfate (De Philippis et al. 2011). These chemical groups in EPS can effectively bind with heavy metal ions through ions exchange or complexation (Gong et al. 2005; Fang et al. 2011).

Clay is a main component in sediment (Hou et al. 2013). Previous studies indicated that bacterial EPS could be absorbed by clays and sediments through hydrogen bonding and some other chemical bondings (Pierre et al. 2014; Cao et al. 2011; Fang et al. 2012). EPS addition changed the adsorption characteristics of heavy metal ions by clays, which in turn changed the concentration of heavy metal ions in water (Fang et al. 2010). The major part of heavy metal ions in aquatic environment is deposited in sediment through precipitation, sorption and complexation. The deposition of heavy metal ions from water column to sediment would decrease their concentration in water, and then decrease their bio-toxicity, and vice versa. Thus, studying on the transfer of heavy metal ions between water–sediment systems is crucial in evaluation of the ecological effect and the health risk of heavy metal contamination in aquatic environment.

The structures of EPS originated from different bacteria are different (Pereira et al. 2009). Though the effects of some bacterial EPS, e.g. originated from *Pseudomonas putida*, *Bacillus subtilis*, on the adsorption characteristics of heavy metal ions onto clays were studied (Fang et al. 2010; Mikutta et al. 2012), the effects of the EPS, originated from bloom-forming cyanobacteria, on the adsorption characteristics of heavy metal ions by clays remain unknown. In order to elucidate the transfer characteristics of Cd(II) in eutrophic water received cyanobacterial blooms, the effects of EPS originated from *Microcystis* on the adsorption and desorption characteristics of Cd(II) by kaolinite and their possible mechanisms were investigated in this study.

## Methods

### Cyanobacterial strain, culture conditions, EPS extraction, and reagents

Bloom-forming cyanobacterial strain *M*. *aeruginosa* NIES-843 was originated from the National Institute of Environmental Science, Japan, and was kindly provided by Professor Renhui Li (Chinese Academy of Sciences). *M*. *aeruginosa* NIES-843 was grown axenically in CT medium (Ichimura 1979) at 25 ± 1 °C under a photoperiod cycle of 12:12 light/dark. The light intensity was set as 30 μmol photons/(s m^2^). The cell free cultures of *M*. *aeruginosa* NIES-843 were collected at stationary phase by centrifuge at 10,000×*g* for 10 min. The EPS in the cultures was purified in deionised water (18 MΩ cm) using dialysis bags (1000-Da cutoff). The purified EPS solutions were dried using vacuum freezer, and then stored at −20 °C. CdCl_2_·2.5H_2_O and other reagents used in this study were purchased from Sinopharm Group Chemical Reagent Ltd. (Shanghai, China), and were of analytical grade.

### Preparation of kaolinite

Kaolinite was purchased from Shanghai 54 Chemical Reagent Ltd (Shanghai, China), and it was further purified by washing with ethanol for 3 times, and then followed by washing with deionised water (18 MΩ cm) for 3 times. The fractions of kaolinite, less than 2 μm, were prepared according to the method described by Cai et al. (2006).

## Adsorption experiments and adsorption isotherm

Adsorption experiments were carried out in 10 mL centrifuge tube containing appropriate volume of deionised water (18 MΩ cm), 30 mg of kaolinite or the composite of kaolinite (30 mg) and EPS. The suspensions of kaolinite and the composite of kaolinite + EPS were incubated on a shaker for 30 min with a speed of 120 rpm, and then appropriate mount of Cd(II) and supporting electrolyte (KNO_3_, final concentration 0.01 M) were added into centrifuge tube, and the total volume was brought to 6 mL using deionised water. The centrifuge tubes were agitated on a shaker at a speed of 120 rpm for 4 h (reached equilibrium). The pH value was set as 7 except pH experiments, and the temperature was set as 25 °C except temperature experiments. In order to study the effect of EPS concentration on the adsorption of Cd(II) by kaolinite, the final EPS concentration was set as 0.1, 0.3, 0.6, 1, 2, and 3 g/L, and the initial Cd(II) concentration was set as 5 mg/L. In pH experiments, the pH value was set as 5, 6, 7, and 8, respectively, and the initial Cd(II) concentration was also set as 5 mg/L. For determination of the effect of initial Cd(II) concentration on its adsorption by kaolinite and the composite of kaolinite + EPS, the initial Cd(II) concentration was set from 5 to 500 mg/L, and the final EPS concentration in the treatment of kaolinite + EPS was set as 0.6 g/L. In temperature experiments, the temperature was set as 20, 25, 30, 35, and 40 °C, respectively. After equilibrium, the suspensions were centrifuged at 12,000×*g* for 10 min, and the Cd (II) in the supernatant was determined using atomic absorption spectrometer (Varian Techtron Pty. Ltd., Victoria, Australia). The amount of adsorbed Cd(II) was calculated from the differences between the initial Cd(II) concentration and the residual concentration after sorption. In order to study the adsorption isotherm of Cd(II) by kaolinite and the composite of kaolinite + EPS, adsorption data were fitted using Langmuir model and Freundlich model in linear form (Eqs. 1 and 2), respectively.

1 $$\frac{1}{{q_{e} }} = \frac{1}{{q_{\text{max} } K_{L} C_{e} }} + \frac{1}{{q_{\text{max} } }}$$

2 $$\ln q_{e} = \ln K_{f} + \frac{1}{n}\ln C_{e}$$

where *q*_*e*_ is the amount of adsorbate absorbed by adsorbent, *C*_*e*_, the equilibrium concentration, *q*_max_, the maximum adsorption capacity upon monolayer saturation adsorbent, *K*_*L*_, the constant related to the adsorption energy, and the *K*_*F*_ and n are Freundlich parameters involved in the relative adsorption capacity and the affinity between adsorbent and adsorbate, respectively.

### X-ray diffraction and Fourier transform infrared spectroscopy analysis

The crystal structures of kaolinite and the composite of kaolinite + EPS were recorded using a XRD-6000 instrument (Shimadzu Seisakusho Ltd., Japan) employing graphite monochromatized Cu K*α* radiation, with scanning rate of 4°/min and ranging from 5° to 75°. Fourier transform infrared (FT-IR) spectra of kaolinite and the composite of kaolinite + EPS were obtained on a spectrometer (PerkinElmer Spectrum 65, Perkin-Elmer Co., Norwalk, CT, USA).

### Desorption of Cd(II)

Desorption of Cd(II) from the kaolinite and the composite of kaolinite + EPS was performed using deionised water or NH_4_NO_3_ or EDTA as desorbent according to the methods previously described by Fang et al. (2011).

### Statistical analysis

Statistical analysis was done by one-way ANOVA using SPSS (version 13.0, SPSS Inc., Chicago, IL, USA). Difference was considered to be significant at *P* < 0.05 (LSD).

## Results

### Effects of EPS on the adsorption of Cd(II) by kaolinite

**Fig. 1:**
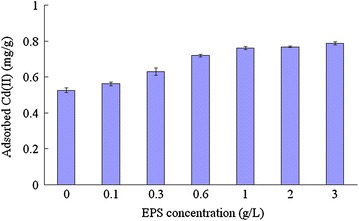
Effects of cyanobacterial EPS on the adsorption of Cd(II) by kaolinite. Data are presented as average value ± standard deviation (n = 3)

As indicated in Fig. 1, the EPS had a positive effect on the adsorption of kaolinite toward Cd(II). With the increase of EPS addition, the adsorbed Cd(II) increased. Compared with the control, the amount of adsorbed Cd(II) increased 6.95, 19.68, 36.85, and 44.60 % at the EPS addition level of 0.1, 0.3, 0.6, and 1 g/L, respectively. The positive effects of EPS on the adsorption of Cd(II) by kaolinite reached plateau phase at the addition level of 1 g/L.

### Characteristics of Fourier transform infrared spectroscopy

**Fig. 2:**
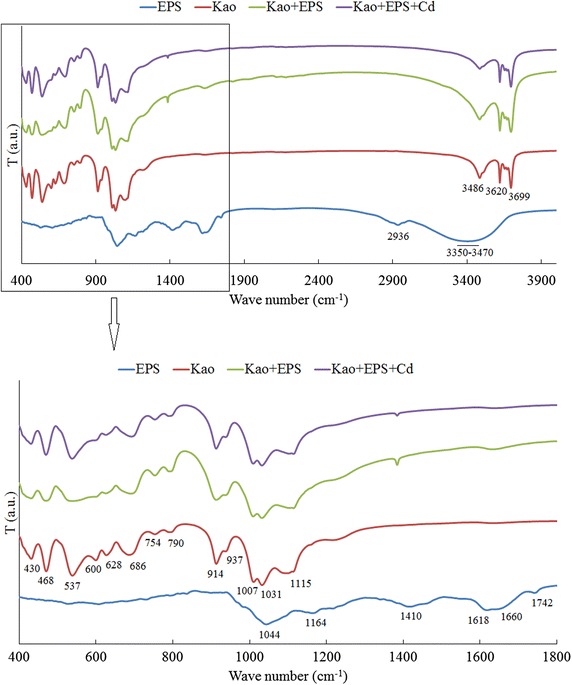
FT-IR spectra of kaolinite, EPS, and the composite of kaolinite + EPS before and after Cd(II) adsorption. Kao: Kaolinite; Kao + EPS: the composite of kaolinite + EPS before Cd(II) adsorption; Kao + EPS + Cd: the composite of kaolinite + EPS after Cd(II) adsorption

The Vibrational spectra of EPS, kaolinite and the composite of kaolinite + EPS before and after Cd(II) adsorption, are shown in Fig. 2. The main absorption bands of EPS were corresponding to phosphorylated compounds (1044 cm^−1^), ring vibrations of polysaccharides (1164 cm^−1^), COO^−^ groups (1410 and 1618 cm^−1^), C=O of amides (1660 cm^−1^), C=O of RCOOR (1742 cm^−1^), C-H (2936 cm^−1^), and O–H (3350-3470 cm^−1^), respectively. Typical frequency band corresponding to Si–O (1115, 1031, 1007 cm^−1^), Al-O (430 and 643 cm^−1^), and O–H (3486, 3620 and 3699 cm^−1^) presented in kaolinite and the composite of kaolinite + EPS. No matter Cd(II) adsorption or not, the spectral features of the composite of kaolinite + EPS were exhibited a same pattern.

### Characteristics of X-ray diffraction

**Fig. 3:**
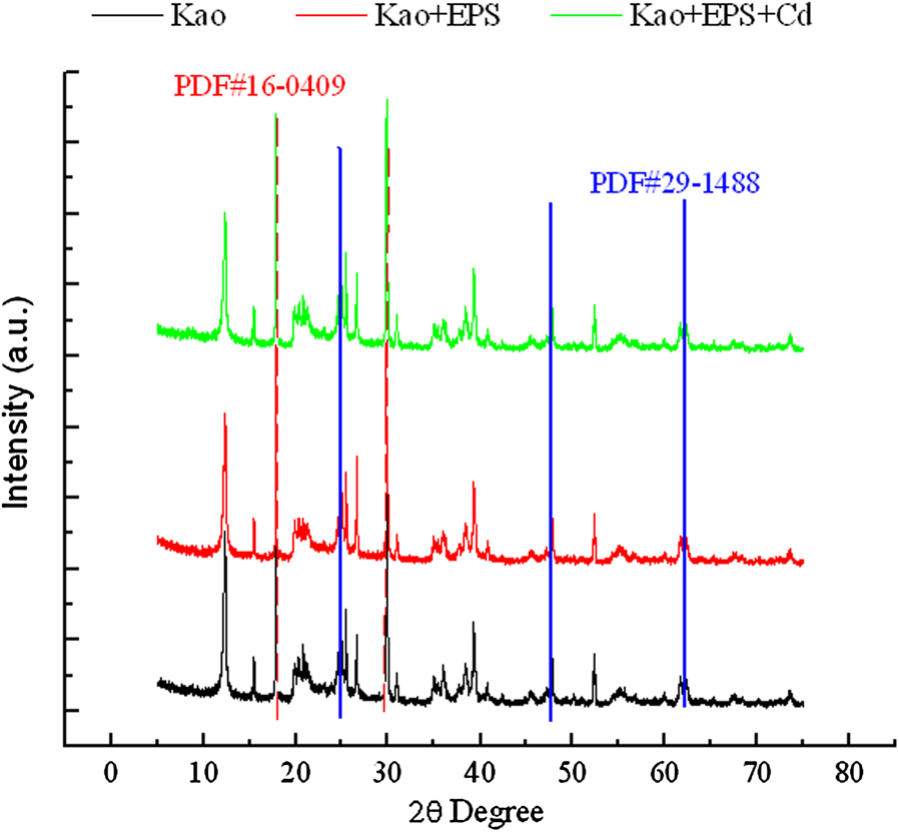
XRD patterns of kaolinite and the composite of kaolinite + EPS before and after Cd(II) adsorption

Figure 3 shows the characteristics of X-ray diffraction of different treatments. The XRD pattern of kaolinite was similar with that of the composite of kaolinite + EPS. Typical diffraction peaks indexed as PDF#29-1488 and PDF#16-0409 for kaolinite presented in all treatments. EPS addition and Cd(II) adsorption did not change the diffraction patterns of kaolinite.

### Effect of pH on the adsorption characteristics

**Fig. 4:**
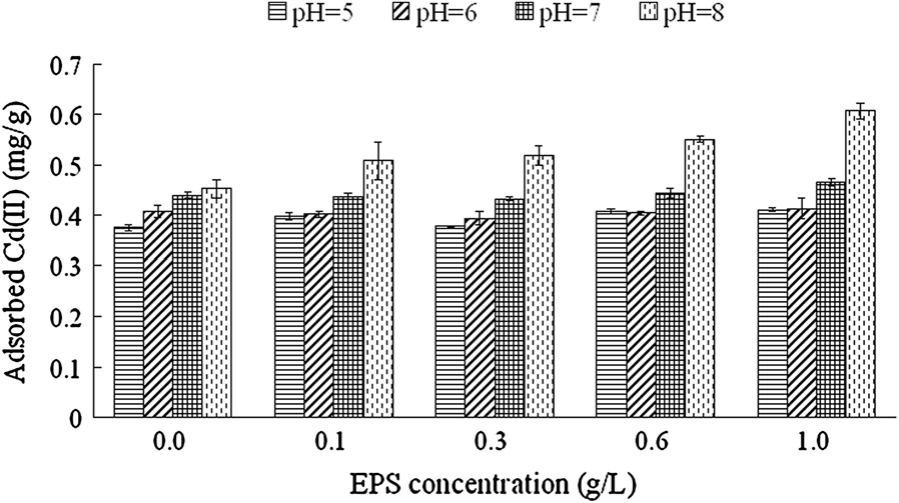
Effects of pH on the adsorption of Cd(II) by kaolinite and the composite of kaolinite + EPS. Data are presented as average value ± standard deviation

The adsorptions of Cd(II) by kaolinite and the composite of kaolinite + EPS were all significantly influenced by the pH value in the adsorption system (Fig. 4). With the increase of pH from 5 to 8, the adsorptions of Cd(II) by all treatments continue to increase. EPS and pH value has a synergistic effect on the adsorption of Cd(II) onto kaolinite. Compared with the amount of adsorbed Cd(II) at pH 5, the adsorbed Cd(II) by kaolinite increased 20.6 % at pH 8, whereas it increased 47.5 % under the condition of EPS addition at the level of 1 g/L under this pH value.

### Different initial Cd(II) concentration and adsorption isotherm

**Fig. 5:**
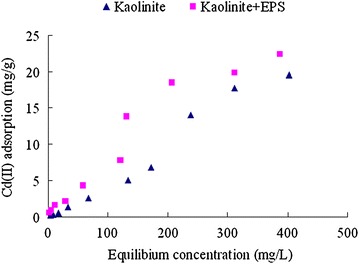
Effects of initial Cd(II) concentration on its adsorption onto kaolinite and the composite of kaolinite + EPS. Data are presented as average value ± standard deviation

**Fig. 6:**
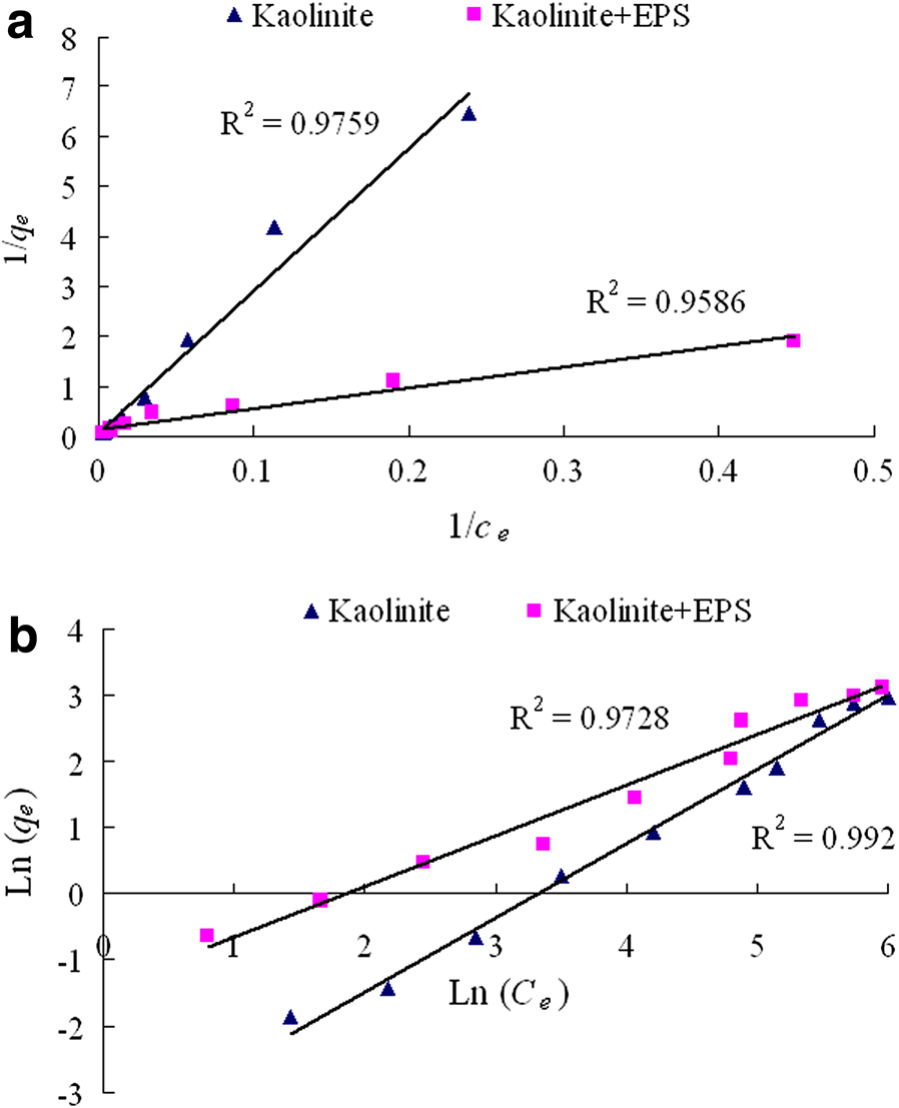
Langmuir (**a**) and Freundlich (**b**) isotherm plots for Cd(II) adsorption by kaolinite and the composite of kaolinite + EPS

The adsorption of Cd(II) by kaolinite and the composite of kaolinite + EPS all increased with the increase of initial Cd(II) concentration (Fig. 5). The adsorption of Cd(II) by the composite of kaolinite + EPS was higher than that by kaolinite under the condition of a same initial Cd(II) concentration. In order to study the adsorption isotherm of Cd(II) by kaolinite and the composite of kaolinite + EPS, the adsorption data were fitted by Langmuir model and Freundlich model. As showed in Fig. 6 and Table 1, Both Langmuir model and Freundlich model were all well fitted by the adsorption data of kaolinite toward Cd(II) with a R square of 0.976 and 0.992, respectively. As the adsorption of Cd(II) by the composite of kaolinite + EPS, the R square is 0.958 for Langmuir model and 0.972 for Freundlich model.

**Table 1:** Parameters of Langmuir model and Freundlich model for the adsorption of Cd(II) onto kaolinite and the composite of kaolinite + EPS

	Langmuir model			Freundlich model		
	*q*_*max*_ (mg/g)	*K*_*L*_	R^2^	*n*	*K*_*F*_	R^2^
Kaolinite	18.42	0.002	0.976	0.89	0.02	0.992
Kaolinite + EPS	7.59	0.032	0.959	1.31	0.24	0.973

### Effect of temperature on adsorption characteristics

**Fig. 7:**
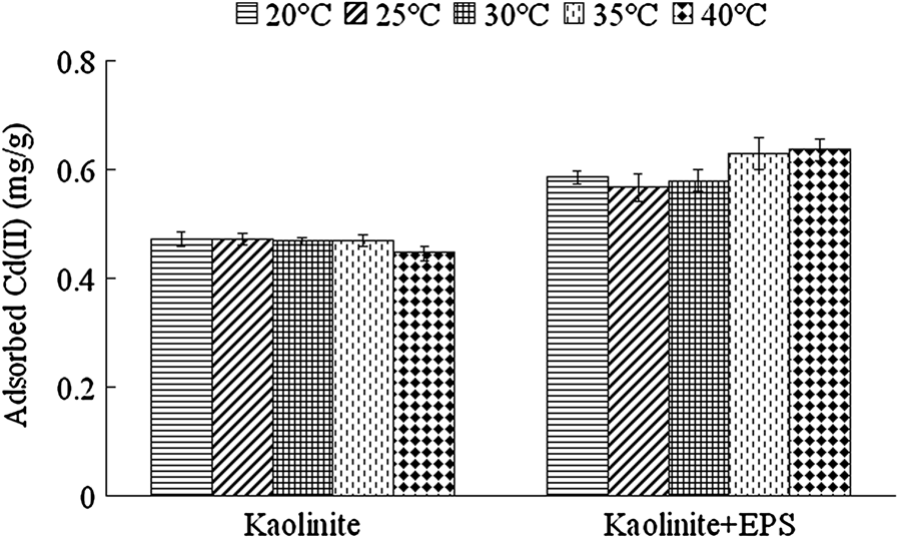
Effects of temperature on the adsorption of Cd(II) by kaolinite and the composite of kaolinite + EPS. Data are presented as average value ± standard deviation

The effects of temperature on the adsorption of Cd(II) by kaolinite and the composite of kaolinite + EPS are shown in Fig. 7. The adsorption of Cd(II) by kaolinite was not obviously influenced by the temperature at a range from 20 to 35 °C. However, the amount of adsorbed Cd(II) at 40 °C decrease 5–6 % when compared with those at 20–35 °C. As for the composite of kaolinite + EPS, the adsorption toward Cd(II) was not obviously influenced at a range from 20 to 30 °C, whereas it increased 7.4 % at 35 °C and 8.7 % at 40 °C when compared with that at 20 °C.

### Desorption characteristics

**Fig. 8:**
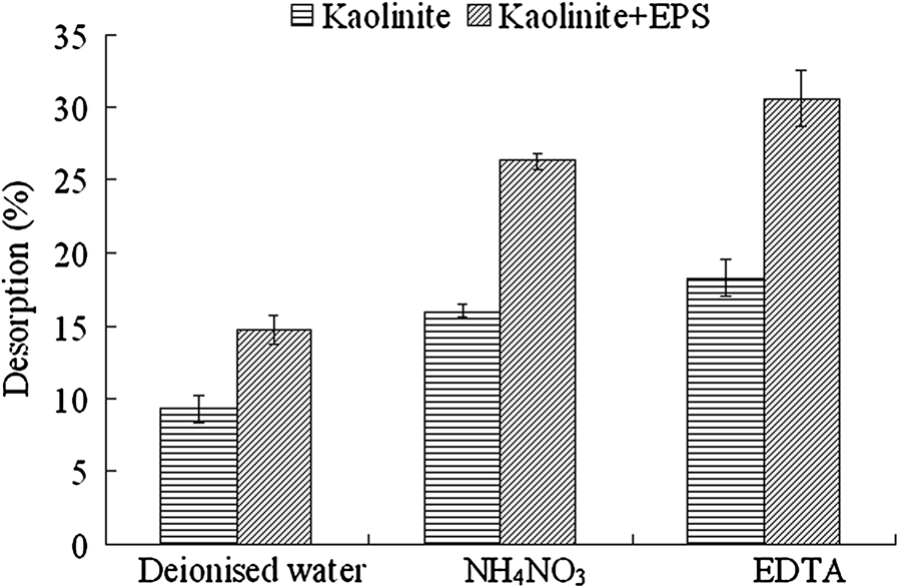
Desorption ratio of Cd(II) from kaolinite and the composite of kaolinite + EPS using different desorbents. Data are presented as average value ± standard deviation

Figure 8 shows the percentage of Cd(II) desorbed from kaolinite and the composite of kaolinite + EPS with different desorbents. Deionised water desorbed 9.31 and 14.72 % of Cd(II) from kaolinite and the composite of kaolinite + EPS, respectively. The desorption ratio for NH_4_NO_3_ treatment was 16.03 % for kaolinite and 26.31 % for the composite of kaolinite + EPS. The desorption ratio of Cd(II) by EDTA was 18.32 % for kaolinite and 30.60 % for the composite of kaolinite + EPS.

## Discussion

**Fig. 9:**
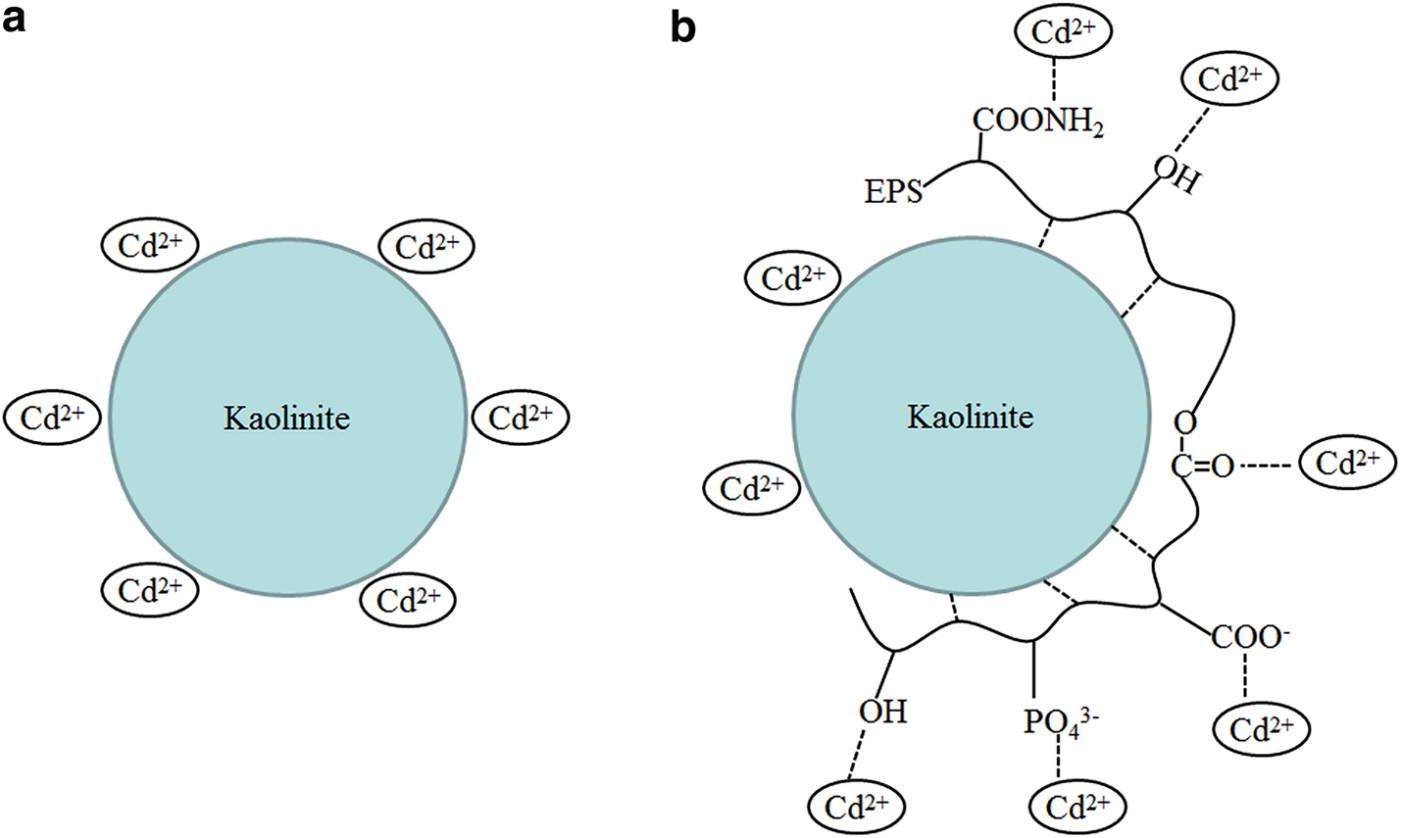
Schematic diagram for the adsorption of Cd(II) by kaolinite (**a**) and the composite of kaolinite + EPS (**b**)

The results in this study showed that the EPS originated from *M. aeruginosa* increased the adsorption capacity of kaolinite toward Cd (II), and this positive effect increased along with the increase of EPS concentration in solution. Similar results were reported by Fang et al. (2010), who found that the composite of montmorillonite and EPS (from *Pseudomonas putida*) showed higher adsorption capacity toward Cu(II) than pure montmorillonite. Some previous studies showed that bacterial EPS could be absorbed by kaolinite, montmorillonite through hydrogen bonding (Cao et al. 2011; Mikutta et al. 2012). Bacterial EPS enrich hydroxyl groups, carboxyl groups, acetylated amino, and some other negative charged groups (De Philippis et al. 2011). These groups can effectively binding with heavy metal ions. Based on the results of FT-IR determination, a schematic diagram for the mechanism of positive effects of cyanobacterial EPS on the adsorption of Cd(II) by kaolinite were proposed and showed in Fig. 9. The groups like PO_4_^3−^, -COO^−^, -CONH_2_, RCOOR, and –OH on the EPS may response for the positive effects of EPS on the adsorption of Cd(II) by kaolinite.

The interlayer spacing of kaolinite is 0.716 nm. Only small high polar molecules can enter into the interlayer of kaolinite (Tang et al. 2015). The EPS are large bio-molecules. The size of EPS is far larger than the interlayer spacing of kaolinite. Results of XRD determination showed that cyanobacterial EPS addition and Cd(II) adsorption did not affect the diffraction patterns of kaolinite, indicating that the EPS and Cd(II) all bound on the surface of kaolinite and not intercalated into the interlayers.

The main absorption bands corresponding to C–O, C=O, and O–H are presented in EPS in this study. They are in consistent with previous studies that polysaccharides are the main constituents of bacterial EPS (Xu et al. 2013). Compared with the vibrational spectra of kaolinite + EPS before Cd(II) adsorption, no new absorption band was found after Cd(II) adsorption for this treatment. However, we also noted that the vibration intensity of the band corresponding to –OH was strong in the treatment of kaolinite + EPS before Cd(II), but it became weak after Cd(II) adsorption. Thus, we deduced that hydroxyl groups were involved in the adsorption of Cd(II) by the composite of kaolinite + EPS.

Langmuir model is known as monolayer sorption, while the Freundlich model is suitable to multilayer sorption (He and Chen 2014). Our results indicated that both Langmuir model and Freundlich model were all well fitted by the data originated from the adsorption of Cd(II) by kaolinite, and the deduced *q*_max_ from Langmuir model was in consistent with experimental data. However, the adsorption isotherm of Cd(II) by the composite of kaolinite + EPS was only suitable to Freundlich model but not Langmuir model since the deduced *q*_max_ from Langmuir model was far lower than experimental data. Thus, we deduce that the addition of cyanobacterial EPS increased the heterogeneity on the surface of kaolinite. The parameter *n* from Freundlich model reflects the affinity between adsorbent and adsorbate. The value of *n* for the composite of kaolinite + EPS is higher than that of pure kaolinite, suggesting that the composite of kaolinite + EPS has higher affinity toward Cd(II) than pure kaolinite.

As for the thermodynamics of the adsorption of Cd(II) by kaolinite, previous studies gave complex and contradictory conclusions. For example, Sari and Tuzen (2014) reported that the adsorption of Cd(II) onto kaolinite was an exothermic reaction while it was described as an endothermic reaction by Angove et al. (1998). Results in this study supported the conclusion that it was an exothermic reaction since the increase of temperature decreased the adsorption of Cd(II) by kaolinite. As for the composite of kaolinite + EPS, the adsorption of Cd(II) by this composite increased along with the increase of temperature, and it exhibited as an endothermic process. The adsorptions of EPS toward Pb(II) and Zn(II) were reported as endothermic processes (Wang et al. 2013). Thus, we deduced that the adsorption of EPS on the surface of kaolinite response for the shift from exothermic process (kaolinite) to endothermic process (composite of kaolinite + EPS).

The fractions of Cd(II) desorbed by deionised water corresponding to physical adsorption ones (Fang et al. 2011). Ammonium nitrate could release the part of Cd(II) adsorbed by physical adsorption and ion exchange (Brady and Tobin 1995). EDTA could release the part of Cd(II) adsorbed through physical adsorption, ion exchange, and the complexation with carboxylic groups, acetylated amino and phosphate (Volesky and Holan 1995; Li et al. 2010). Compared with the treatment of pure kaolinite, the ratio of desorbed Cd(II) by H_2_O, NH_4_NO_3_, and EDTA all increased in the treatment of kaolinite + EPS, suggesting that EPS addition increased the adsorption of Cd(II) by kaolinite through physical adsorption, ion exchange, and complexation.

## Conclusion

Cyanobacterial EPS bound on the surface of kaolinite. The composite of kaolinite + EPS showed higher adsorption capacity toward Cd(II) than pure kaolinite, and hydroxyl groups were involved in the adsorption process. The addition of cyanobacterial EPS increased the heterogeneity on the surface of kaolinite, and change the thermodynamics from exothermic process to endothermic one.
